# The role and clinical applications of exosomes in cancer drug resistance

**DOI:** 10.20517/cdr.2024.97

**Published:** 2024-11-04

**Authors:** Wenxuan Pan, Qun Miao, Wenqian Yin, Xiaobo Li, Wencai Ye, Dongmei Zhang, Lijuan Deng, Junqiu Zhang, Minfeng Chen

**Affiliations:** ^1^State Key Laboratory of Bioactive Molecules and Druggability Assessment, Jinan University, Guangzhou 510632, Guangdong, China.; ^2^College of Pharmacy, Jinan University, Guangzhou 510632, Guangdong, China.; ^3^School of Traditional Chinese Medicine, Jinan University, Guangzhou 510632, Guangdong, China.; ^#^Authors contributed equally.

**Keywords:** Exosome, drug resistance, implications for clinical treatment

## Abstract

Tumor-secreted exosomes are heterogeneous multi-signal messengers that support cancer growth and dissemination by mediating intercellular crosstalk and activating signaling pathways. Distinct from previous reviews, we focus intently on exosome-therapeutic resistance dynamics and summarize the new findings about the regulation of cancer treatment resistance by exosomes, shedding light on the complex processes via which these nanovesicles facilitate therapeutic refractoriness across various malignancies. Future research in exosome biology can potentially transform diagnostic paradigms and therapeutic interventions for cancer management. This review synthesizes recent insights into the exosome-driven regulation of cancer drug resistance, illuminates the sophisticated mechanisms by which these nanovesicles facilitate therapeutic refractoriness across various malignancies, and summarizes some strategies to overcome drug resistance.

## INTRODUCTION

Despite advancements in early detection, precision medicine, and targeted therapies, a substantial proportion of patients still develop resistance to chemotherapeutic agents, which often hinders successful treatment outcomes and compromises patient survival across multiple cancer types. Millions of new cancer cases and deaths occur annually, emphasizing the pressing need to understand and overcome this issue^[1,2]^. In recent years, increasing evidence has indicated that substantial cargos of information are released from cells via lipid bilayer-enclosed vesicles typically termed exosomes and microvesicles. It has been demonstrated that these vesicles are closely associated with drug resistance^[3]^.

Exosomes are endosomal-derived nanoscale extracellular vesicles (EVs) that have become important players in the intricate interactions between cancer cells and their surroundings. These vesicles are released by both tumor and stromal cells and transport a variety of bioactive substances, such as proteins, lipids, and non-coding RNAs (ncRNAs), which can dramatically alter the phenotypic and functional traits of recipient cells and foster a milieu of therapeutic evasion. Exosomes have been shown to contribute to resistance against chemotherapeutic, hormonal, and targeted therapies, as well as to shape the immune-suppressive microenvironment, promoting cancer progression and metastasis^[4-6]^.

The details of the mechanisms by which resistance appears to occur are outlined. Exosomes from drug-resistant cancer cells can reduce drug accumulation by interacting with chemotherapeutic agents, limiting their cytotoxic effects^[4]^, facilitating the transfer of functional proteins and ncRNAs, activating pro-survival signaling pathways, such as epithelial-mesenchymal transition (EMT), and enhancing chemotherapy resistance^[7,8]^. Moreover, exosomes can mediate the transfer of drug resistance traits from resistant to sensitive cells, as exemplified by the transfer of miR-155 via exosomes, which promotes chemoresistance in recipient cancer cells^[9,10]^. Exosomes also modify the tumor microenvironment (TME) by delivering immunosuppressive factors, such as TGF-β and Fas ligands, triggering immune cell apoptosis or recruiting myeloid-derived suppressor cells (MDSCs), thus dampening antitumor immune responses^[11-13]^. As previously stated, the multifaceted function of exosomes in cancer drug resistance underscores their capacity as prognostic and surveillance indicators for treatment outcomes. The unique exosomal cargo profiles observed in drug-resistant cells and their ability to reflect the dynamic changes in response to therapy make them valuable candidates for liquid biopsy-based diagnostics^[10]^.

The secretion of exosomes is an important way to influence the behavior of cancer cells (and vice versa). This review focuses on the role of exosomes in modulating drug resistance by influencing the different facets of the TME. We aspire to consolidate the current understanding of the underlying mechanisms, elucidate how exosomes contribute to the process of resistance, and subsequently outline potential strategies that could attenuate or reverse this resistance, thereby contributing to the advancement of precision oncology.

## EXOSOMES

**Figure 1:**
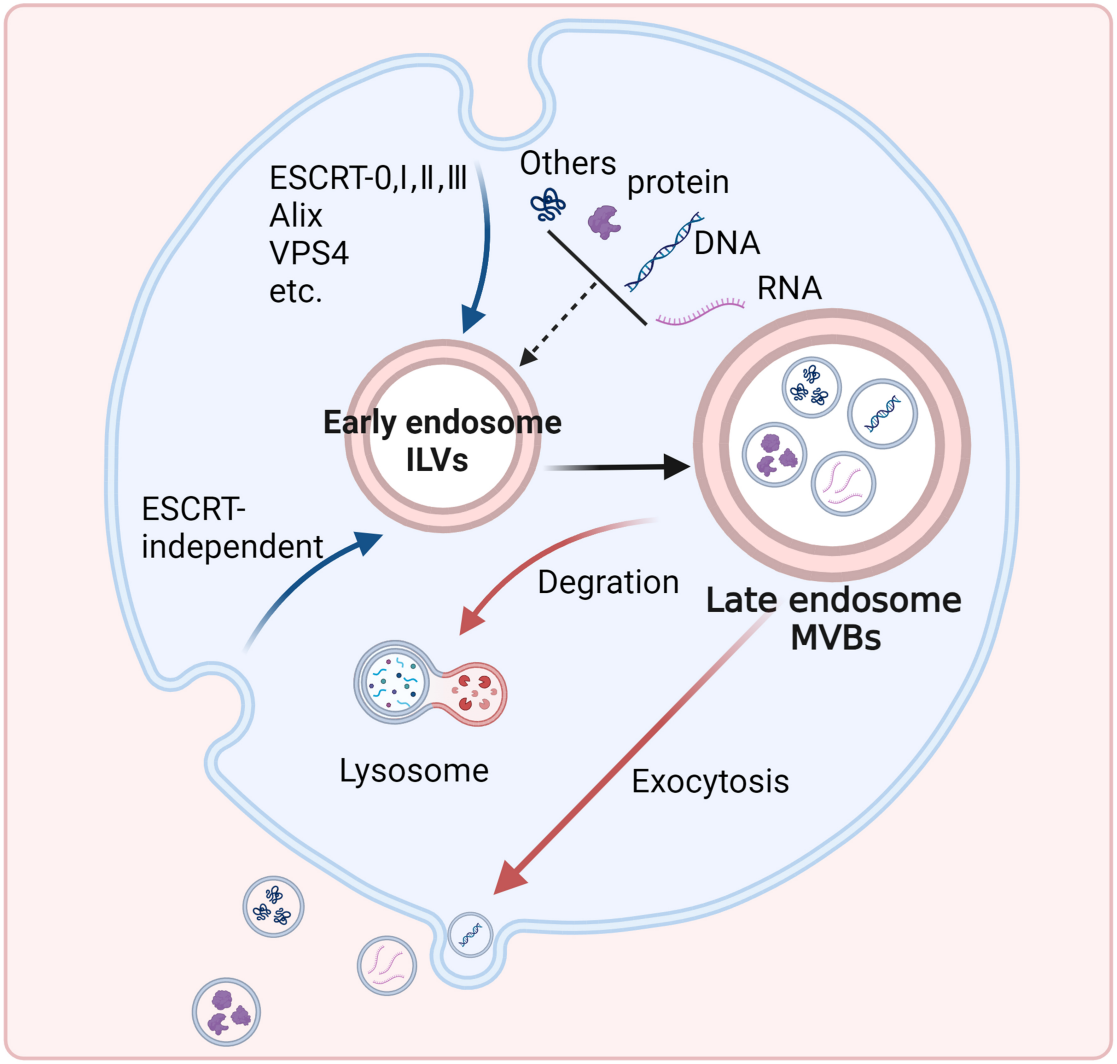
Schematic representation of the exosome production process. The biogenesis of exosomes is linked to the endosomal pathway, which begins with the creation of ILVs within MVBs through two mechanisms, and formed MVBs can fuse with lysosomes or the plasma membrane for different fates. ILVs: Intraluminal vesicles; MVBs: multivesicular bodies.

Exosomes, a subset of EVs^[14-16]^, these nanoscale lipid membrane-enclosed vesicles, typically 30 to 150 nm in diameter, are secreted by most eukaryotic cells and found in various bodily fluids^[17,18]^. Exosome biogenesis involves the inward budding of the endosomal membrane, creating intraluminal vesicles (ILVs) within multivesicular bodies (MVBs)^[18,19]^. The endosomal sorting complex required for transport is a complex with membrane-severing activity that plays a major role in many membrane remodeling processes, including endosomal trafficking, nuclear envelope organization, and cytokinesis. The process of exosome biogenesis is governed by the endosomal sorting complexes required for transport (ESCRT)-dependent pathways^[10,15,20]^. MVBs can degrade with lysosomes or secrete exosomes^[18]^, carrying diverse cargoes^[20-23]^ based on the parent cell’s physiological state, allowing exosomes to transmit specific signals to recipient cells^[24,25]^ [Figure 1].

**Figure 2:**
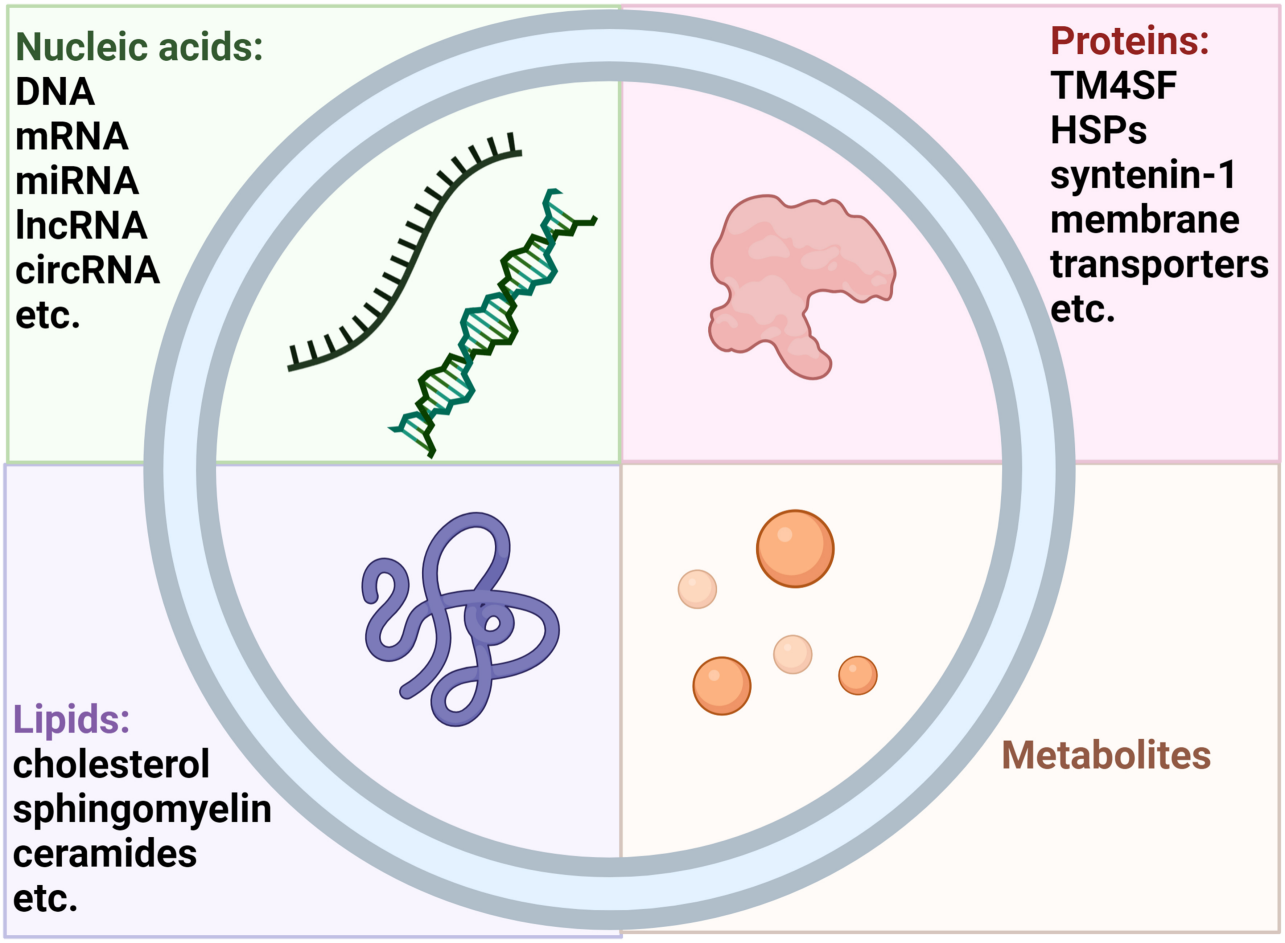
The different types of molecules found in exosomes, including nucleic acids (DNA, mRNA, miRNA, lncRNA, circRNA), proteins (TM4SF, HSPs, syntenin-1, membrane transporters), lipids (cholesterol, sphingomyelin, ceramides), and metabolites. circRNA: Circular RNAs.

Exosomes are essential for both intercellular communication and the pathophysiology of various diseases^[26-28]^. Exosomes contain a variety of materials, including lipids, proteins, nucleic acids, and even metabolites, which can indicate the health or pathology of the cell^[29-33]^. Exosomes, derived from various sources, contain unique proteins^[28,29]^, lipids^[27,34]^, genomic DNA^[29,30]^, ncRNAs^[27,29,30]^, and bioactive metabolites [Figure 2]. They can transmit genetic information between cells, alter gene expression, and transport bioactive metabolites, highlighting their roles in metabolic regulation and disease progression^[34]^. Their stability and targeting capabilities are influenced by these factors. The versatility of exosomal cargo extends to its capacity to convey immunomodulatory signals, as seen by the presence of programmed death ligand-1 (PD-L1) on exosomes from cancer cells, which influences immune evasion mechanisms^[29]^. This highlights the potential of exosomal content for immune surveillance and therapeutic interventions^[27,28]^.

Exosomes, through regulated cell-to-cell communication, play a crucial role in maintaining physiological homeostasis by delivering customized cargo to target cells, such as cancer cells promoting tumor progression and immune cells modulating responses^[35,36]^. The specificity of exosome uptake is achieved through various mechanisms, including surface receptor-ligand interactions and membrane fusion events^[36]^. The study of exosome-mediated communication has far-reaching implications, ranging from understanding fundamental biological processes to the development of novel therapeutic strategies^[36,37]^. Exosomes, as mediators of cell-to-cell communication, shuttle through the TME, are absorbed by surrounding cancer cells or stromal cells, and can transmit information by releasing contents, thereby causing the proliferation, invasion, metastasis and drug resistance of tumor cells.

In summary, exosomes are highly developed messengers that facilitate complex cellular communications. Recent studies have shown that exosomes, as mediators of cell-to-cell drug resistance signaling, play an important role in tumor chemotherapy resistance, metastasis, and immune evasion.

## MECHANISM OF EXOSOMES REGULATING TUMOR DRUG RESISTANCE

**Figure 3:**
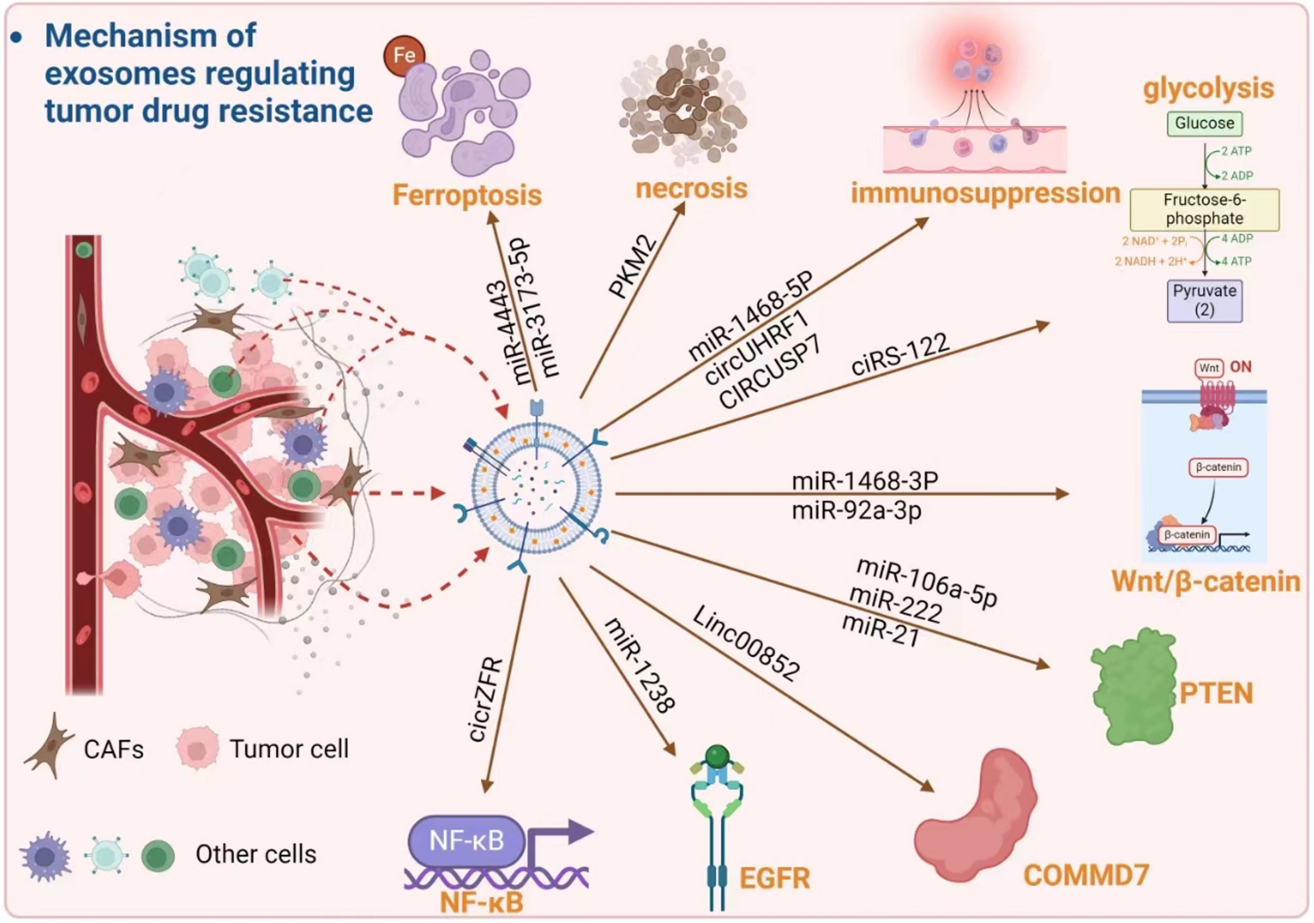
The mechanism of exosomes regulating tumor drug resistance involves many factors and pathways. Exosomes derived from different cells (tumor cells, fibroblasts, and other cells) can regulate different mechanisms by delivering various contents, thus affecting tumor resistance.

Exosomes are essential for coordinating and spreading drug resistance pathways within the TME^[11,38]^. These EVs transfer resistance traits to cells, influencing apoptotic, metabolic, and immune responses, and promoting systemic resistance^[39-41]^. They also contribute to cancer cell chemoresistance by targeting the survival, proliferation, and drug response pathways^[42,43]^ [Figure 3].

Targeting exosomal communication, particularly the exchange of resistance-inducing factors, represents an unexplored avenue for therapeutic intervention^[44]^. Understanding the specific roles and mechanisms of exosomal cargoes in different cancer types holds promise for developing novel strategies to overcome therapeutic resistance. By interrupting these exosome-mediated pathways, researchers have aimed to sensitize tumors to conventional treatments, thereby revitalizing the efficacy of chemotherapy and immunotherapy in cancer management.

### Exosomes derived from tumor cells regulate tumor drug resistance

Tumor-derived exosomes (TDEs) have emerged as key mediators in the establishment and propagation of drug resistance, a phenomenon that poses a substantial challenge to cancer therapy^[45-53]^. These EVs, harboring a cargo of proteins, lipids, and nucleic acids, including miRNAs and lncRNAs, can transfer resistance traits to neighboring sensitive cells or even distant sites, fostering a systemic resistant phenotype^[46,50,52,54,55]^.

In non-small cell lung cancer (NSCLC), exosomes transfer miR-4443, which induces chemotherapy resistance by regulating ferroptosis-related gene expression^[45]^. Another study highlighted the role of exosomal PKM2 in transmitting cisplatin resistance from hypoxic NSCLC cells to sensitive cells, implicating an altered cellular metabolism in the resistance mechanism^[56]^. Similarly, exosomes from hypoxic glioma cells harboring a specific fusion gene were found to disseminate pro-oncogenic signals, including miR-106a-5p, contributing to temozolomide (TMZ) resistance^[46]^. In gastric cancer, exosomes from paclitaxel-resistant cells were shown to enrich miR-155-5p, which was transferred to sensitive cells, inhibiting apoptosis and mediating resistance by suppressing GATA3 and TP53INP1^[51]^. Exosomes from adriamycin-resistant breast cancer cells were found to carry miR-222-5p, which promoted macrophage M2 polarization via phosphatase and tensin homolog (PTEN)/Akt inhibition, fostering a tumor-permissive microenvironment and drug resistance^[49]^. Similarly, exosomal miR-9-5p from tamoxifen-resistant breast cancer cells was demonstrated to be transferred to sensitive cells, where it downregulated adiponectin (ADIPOQ), thereby contributing to drug resistance and enhanced tumor growth^[48]^. In glioblastoma (GBM), exosomal miR-1238 mediates the acquired resistance of GBM cells to TMZ by directly targeting the CAV1/EGFR pathway and affecting the activation of the PI3K-AKT-mTOR signaling pathway^[47]^, while exosomal circUHRF1 was found to induce natural killer cell exhaustion and contribute to anti-PD1 therapy resistance^[57]^.

Moreover, exosomes derived from pancreatic cancer cells were shown to induce apoptosis in CD8^+^ T cells, thereby suppressing immune surveillance and facilitating tumor growth^[55]^. Similarly, exosomes from NSCLC cells carry circUSP7 and modulate the miR-934/SHP2 axis, leading to CD8^+^ T cell dysfunction and resistance to anti-PD1 therapy^[54]^. In gastric cancer, exosomes contribute to the immunosuppressive milieu by suppressing CD8^+^ T cells and natural killer cells while expanding regulatory T cells and MDSCs^[58]^.

TDEs, enriched with PD-L1, can engage with PD-1 on T cells, dampening immune responses and fostering tumor resistance to PD-1/PD-L1 blockade therapies. Strategies targeting exosomal PD-L1 could enhance immunotherapy outcomes^[59]^. Recent studies have also explored the potential of exosomes as carriers for immune checkpoint inhibitors, suggesting their utility in improving cancer treatment efficacy^[60]^. Additionally, an innovative approach using curvature-sensing peptides to disrupt TDEs has shown promise in boosting cancer immunotherapy by inhibiting TDEs-mediated immunosuppression and reshaping the TME^[61]^.

These findings collectively underscore the importance of TDEs in mediating crosstalk between cancer cells and their microenvironment, highlighting exosome-mediated drug resistance as a complex and multifaceted phenomenon that spans different cancer models. Understanding the specific cargo and its targets within exosomes could pave the way for novel therapeutic interventions aimed at overcoming drug resistance in cancer therapy.

### Exosomes from cancer-associated fibroblasts regulate tumor resistance

Cancer-associated fibroblasts (CAFs) have been identified as pivotal contributors to the acquisition of chemoresistance in cancer cells, primarily through exosome-mediated communication^[62-70]^. Similar to tumor cells, CAFs secrete exosomes loaded with diverse cargoes, including miRNAs, lncRNAs, and proteins, that orchestrate resistance mechanisms in neighboring cancer cells^[62-65]^.

Previous studies have indicated that exosomes derived from gemcitabine-treated CAFs contain elevated levels of miR-148b-3p, which, upon transfer to bladder cancer cells, downregulates PTEN and activates the Wnt/β-catenin pathway, thereby promoting chemoresistance and EMT^[62]^. Similarly, CAFs-derived exosomes carrying miR-106b were found to induce gemcitabine resistance in pancreatic cancer by targeting TP53INP1^[64]^. Another study highlighted that CAF-derived exosomal miR-196a promotes proliferation and drug resistance in head and neck squamous cell carcinoma (HNC) cells by targeting CDKN1B and ING5^[63]^. Additionally, CAFs secrete exosomes that highly express miR-3173-5p, which inhibits ferroptosis by targeting ACSL4, thereby promoting gemcitabine resistance in pancreatic cancer cells^[66]^. Moreover, exosomes from CAFs facilitate colorectal cancer (CRC) metastasis and chemoresistance by enhancing stemness and EMT through miR-92a-3p transfer, further validating the role of exosomal miRNAs in these processes^[71]^. Furthermore, CAF-derived exosomes transfer lncRNA CCAL, which upregulates glycolytic pathways and STAT3/NF-κB signaling, promotes chemoresistance, and emphasizes the relevance of ncRNA content in exosomes in tumor progression^[68]^.

Additionally, proteins and other activated materials in CAF-derived exosomes are crucial. For example, studies have demonstrated that CAFs secrete exosomes loaded with cytokines and chemokines, such as IL-6 and CXCL1, which activate the STAT3/NF-κB pathway, contributing to cisplatin resistance in esophageal squamous cell carcinoma (ESOC) and gastric carcinoma (GC)^[67]^. An important component of pancreatic ductal adenocarcinomas, CAFs, release exosomes that improve chemoresistance in recipient cancer cells by expressing Snail. They also exhibit intrinsic resistance to gemcitabine^[72]^. This observation aligns with findings where exosomes from CAFs have been shown to promote resistance to anti-pyrimidine drugs by suppressing the pyrimidine transporter, ENT2, highlighting the role of exosomal cargo in modulating therapeutic sensitivity^[70]^. Exosomes derived from pancreatic cancer CAFs increase the release of exosomes upon gemcitabine treatment, which in turn induces chemoresistance in pancreatic cancer cells by modulating the expression of Snail and miR-146a. This study highlights the potential of using exosome secretion inhibitors as a combinational approach to improve chemotherapy effectiveness^[72]^. Finally, recent studies have highlighted that exosomes derived from CAFs can significantly influence the immunological landscape within the tumor. These CAF-derived exosomes have been shown to carry immunosuppressive molecules, such as PD-L1, which can dampen the antitumor immune response by interacting with immune checkpoint receptors on T cells. This interaction not only subdues the cytotoxic potential of T cells but also promotes a tolerant environment that fosters tumor resistance to immunotherapies, thereby contributing to the development of therapeutic resistance in cancer^[73]^.

These results highlight the intricate relationship between CAFs and cancer cells, in which exosomes act as carriers for factors that lead to resistance. This suggests that focusing on CAF-derived exosomes may be a useful strategy for cancer therapy that aims to overcome chemoresistance.

### The content of exosomal circular RNA regulates tumor resistance

Circular RNAs (circRNAs), a distinct class of ncRNAs characterized by their covalently closed loop structures, have emerged as crucial regulators in the complex interplay between cancer cells and their microenvironment, particularly in the context of chemotherapy resistance. These circRNAs, packaged within exosomes, act as mediators of intercellular communication and convey messages that influence tumor progression and drug responsiveness^[74-80]^.

For instance, in CRC, hsa_circ_0000338 was found to be differentially upregulated in exosomes from FOLFOX-resistant cells, potentially serving as a biomarker for chemoresistance and exerting dual regulatory roles^[76]^. Similarly, in gliomas, exosomal circ_0072083, enriched under hypoxia, promotes TMZ resistance by sponging miR-421 and enhancing SIRT1 expression^[75]^. Furthermore, the role of exosomal circVMP1 in NSCLC demonstrated its capacity to enhance cisplatin resistance through the miR-524-5p-METTL3/SOX2 axis^[78]^. In neuroblastoma (NB), exosomal circDLGAP4 contributes to drug resistance by regulating the miR-143-HK2 axis^[75]^. The involvement of exosomal circRNAs in prostate cancer (PCa) chemoresistance is exemplified by circ-XIAP, which promotes resistance to docetaxel through the miR-1182/TPD52 pathway^[80]^. Similarly, circSFMBT2 in PCa facilitates resistance to docetaxel by interacting with miR-136-5p and modulating TRIB1^[80]^. Breast cancer research also revealed circ_UBE2D2 in exosomes as a mediator of tamoxifen resistance, whereas in osteosarcoma, exosomal circZNF91 enhances resistance to chemotherapy by sponging miR-23b-3p and upregulating SIRT1^[80]^.

Notably, circRNAs within exosomes can interact with immune checkpoint molecules, thereby influencing the sensitivity of tumors to immunotherapy. For instance, exosomal circRNAs, such as circUHRF1 and circTMEM181, can modulate immune responses by targeting immune checkpoint molecules, leading to resistance to immunotherapies like anti-PD1 in hepatocellular carcinoma (HCC)^[57,81]^.

Exosomal circRNAs play a crucial role in cancer treatment by mediating drug resistance mechanisms. They affect drug efflux, intracellular concentrations, cell cycle progression, apoptosis, invasion, and metabolic reprogramming. These findings from various cancer models highlight the central role of exosomal circRNAs in modulating chemotherapy resistance^[75,80]^, suggesting that targeting these exosome-carried circRNAs could be a novel strategy to overcome treatment failure in cancer therapy. Similarly, some studies highlight the intricate relationship between exosomal circRNAs, immune cell modulation, and the development of resistance to immune checkpoint blockade in cancer therapy. The identification of these mechanisms offers promising avenues for the development of novel therapeutic strategies aimed at overcoming immunotherapy resistance in cancer patients.

### The content of exosomal miRNA regulates tumor resistance

Like circRNAs, tissue-specific regulatory networks of miRNAs and their effects on drug transporters, DNA repair systems, and cell survival pathways have become key factors in regulating drug resistance in cancer^[82]^.

For example, exosome-mediated transfer of miR-3613-5p has been shown to enhance doxorubicin (DOX) resistance in breast cancer cells by inhibiting PTEN, underscoring the importance of miRNA shuttling in modulating treatment outcomes^[83]^. MiR-21, which targets PTEN, is known to play a key role in promoting resistance to multiple drugs in breast and stomach cancers^[82]^. Another example of exosome-mediated miRNA transfer was presented, which used arginine-glycine-aspartic acid (RGD)-modified exosomes to deliver miR-484 to ovarian cancer cells, normalizing the tumor vascular system and making it sensitive to chemotherapy^[84]^. In breast cancer, exosomes can mediate resistance and migration to sensitive cells via miR-155^[85]^, and miR-140-3p can suppress PD-L1 expression in cancer cell-derived exosomes, thereby attenuating chemoresistance induced by DOX^[86]^.

Collectively, these findings illustrate the extensive influence of exosomal miRNAs on drug resistance in various cancer types. Strikingly, engineered exosomes have great therapeutic potential for co-delivering miRNA inhibitors and chemotherapy agents to reverse colon cancer resistance. Specifically, the loading of miR-21 inhibitor oligonucleotides and 5-fluorouracil with exosomes can effectively downregulate miR-21 expression in drug-resistant cells, thereby restoring the sensitivity to drugs^[87]^. By manipulating exosomal miRNA content, it might be possible to restore chemosensitivity, thereby enhancing the efficacy of cancer treatments.

## THE DIAGNOSTIC AND THERAPEUTIC SIGNIFICANCE OF EXOSOMES IN TUMOR DRUG RESISTANCE

**Figure 4:**
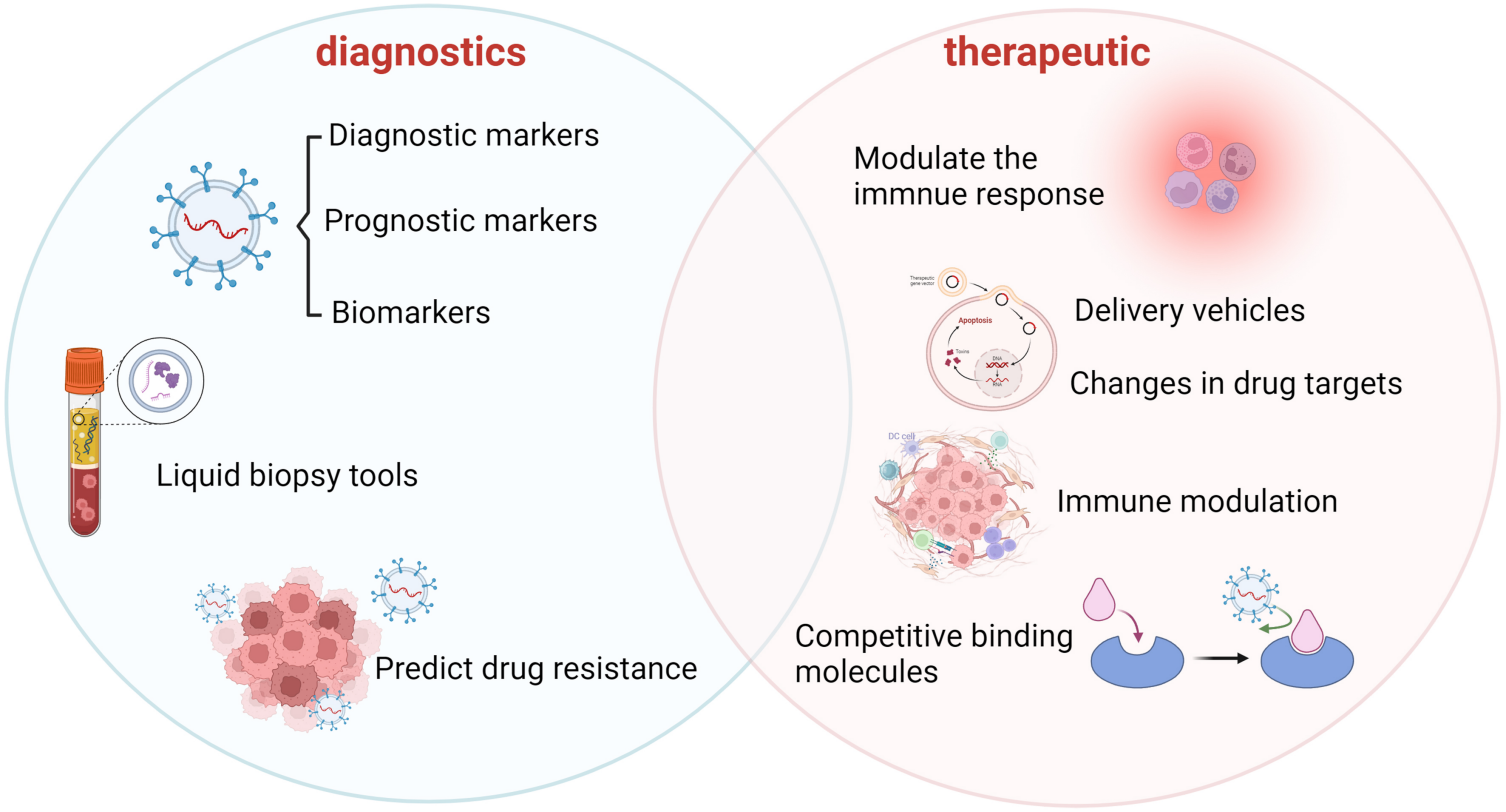
Clinical significance of exosomes in tumor drug resistance: biomarkers, prognostic markers, diagnostic markers, fluid biopsy tools, drug resistance prediction tools, delivery vehicles, changing drug targets, modulating the immune response and competitive binding molecules.

In the realm of cancer research, exosomes have emerged as pivotal players in the development and management of drug resistance, underscoring their diagnostic and therapeutic significance in tumor treatment [Figure 4]. These small EVs, with their unique molecular signatures, are instrumental in intercellular communication, transferring proteins, RNAs, and other biomolecules that can significantly alter the TME and influence therapeutic responses^[88-96]^.

Exosomes are important in the diagnosis and treatment of tumor drug resistance, as they can transfer resistance traits to sensitive cells, promoting resistance by transmitting cargoes, suppressing immune activity, influencing chemotherapeutic efficacy^[92]^, and so on, highlighting the complexity of exosome-mediated resistance in various cancer types.

In NSCLC, exosomes derived from bone marrow mesenchymal stem cells (MSCs) have been shown to enhance chemosensitivity to cisplatin by delivering miR-193a, which targets LRRC1 and reduces drug resistance^[97]^. Conversely, lung cancer cell-derived exosomes (LCCDEs) are implicated in mediating drug resistance through the transfer of resistance-associated proteins and RNA, offering a novel avenue for therapeutic intervention^[98]^.

In HCC, exosomes play a crucial role in drug resistance by transferring molecules that can alter TME and affect drug responsiveness. Strategies targeting exosomes, such as siRNA-mediated silencing of circRNA-SORE, have demonstrated potential in enhancing sorafenib efficacy and overcoming resistance in HCC models^[99,100]^.

Exosomes derived from MSCs have emerged as a promising therapeutic strategy for GBM, a highly aggressive brain cancer, by enhancing the delivery of chemotherapeutic agents and overcoming drug resistance. These nanoscale vesicles can ferry miRNAs that sensitize tumor cells to chemotherapies, thereby offering a novel avenue to combat the refractoriness of brain tumors^[101]^.

PCa treatment resistance is associated with exosomes, as the presence of androgen receptor splice variant 7 (AR-V7) in exosomal RNA correlates with resistance to hormonal therapy. This highlights the potential of exosomal biomarkers in predicting treatment outcomes and personalizing therapeutic strategies for PCa patients^[102,103]^.

Exosomes derived from CAFs play a pivotal role in the chemoresistance of CRC by promoting cancer cell stemness and EMT, which are key factors in therapy resistance. The transfer of exosomal miR-92a-3p from CAFs to CRC cells has been shown to enhance stemness and EMT, thereby contributing to the chemoresistance of CRC to 5-FU/L-OHP regimens. This finding underscores the potential of targeting exosomal components as a novel therapeutic strategy to overcome chemoresistance in CRC^[71,104]^.

In triple-negative breast cancer (TNBC), EVs, including exosomes, play a crucial role in the development of drug resistance by transferring proteins, miRNAs, and other biomolecules that can modulate therapeutic responses. EVs derived from gemcitabine-resistant TNBC cells have been shown to transfer resistance traits to sensitive cells, highlighting their potential as mediators of chemoresistance^[105]^.

Exosomes hold great promise in clinical research for their diagnostic and therapeutic potential in diseases, particularly cancer. They exhibit unique molecular signatures in the bodily fluids of cancer patients, making them valuable for early detection and prognosis assessment. For instance, GPC1-positive exosomes are diagnostic indicators for early-stage pancreatic cancer^[96]^, while exosomal lncRNA SNHG14 levels are associated with trastuzumab resistance in breast cancer^[92]^. The interplay between exosomes and the immune system is crucial for their function in cancer. Exosomes can modulate the immune response by delivering immunosuppressive factors or antigens, as well as by promoting the polarization of immune cells, such as macrophages and T cells, toward protumoral phenotypes^[94,106]^. In contrast, engineered exosomes hold promise for immunotherapy, as they can be designed to express immuno-stimulatory proteins or loaded with cytokines to enhance immune cell activation and antitumor immunity^[106]^.

Exosomes have also emerged as crucial vectors in conveying bioactive molecules, such as proteins, lipids, nucleic acids, and metabolites. For example, exosomes from drug-resistant cancer cells can horizontally transfer miRNAs that confer resistance to neighboring sensitive cells, and lncRNA ARSR within exosomes can competitively bind miR-34 and miR-449 to enhance AXL and MET expression, leading to sunitinib resistance in renal cancer^[88,95]^. This suggests that exosomal contents can shape the therapeutic microenvironment by disseminating resistance traits among cancer populations.

The potential of exosomes as therapeutic targets and diagnostic tools is further emphasized by their inherent ability to traverse biological barriers, including the blood-brain barrier (BBB)^[88]^, making them attractive candidates for delivering therapeutic nucleic acids. For instance, engineered exosomes carrying siRNA against MALAT1 were shown to reduce docetaxel resistance in lung cancer^[91]^, while exosomes loaded with a miRNA cocktail were found to suppress enzalutamide-resistant PCa^[88]^, highlighting the potential for reversing resistance mechanisms.

In summary, the diagnostic and therapeutic significance of exosomes in tumor drug resistance is profound. They serve as biomarkers for early detection and prognosis assessment, modulate the immune response, and convey bioactive molecules that can shape the therapeutic microenvironment. The ability of exosomes to traverse biological barriers further enhances their potential as therapeutic agents. Future research leveraging exosome-mediated intercellular communication and their unique biological properties could unlock new dimensions in precision medicine for cancer treatment and management.

## CONCLUSION

This review has encapsulated the recent advances in our understanding of exosome-mediated regulation of tumor drug resistance, shedding light on the intricate mechanisms by which these nanoscale vesicles contribute to the development and dissemination of therapeutic refractoriness across various cancer types. We have elucidated the pivotal role of exosomes in ferrying a myriad of bioactive molecules, including miRNAs, lncRNAs, circRNAs, and proteins, which act in concert to alter cellular pathways, modulate metabolism, interfere with drug efficacy, and shape the TME in favor of chemoresistance. A key highlight is the recognition of exosomes as conveyors of resistance traits from both tumor cells and CAFs, underscoring the complexity of intercellular communication in fostering treatment-resistant phenotypes.

Despite the progress outlined, several questions persist in the exosome-mediated drug resistance landscape. Above all, the heterogeneity of exosomal cargoes and their differential effects on recipient cells across cancer types pose a significant challenge. Exosomal heterogeneity, stemming from differences in their cellular origins, cargo composition, and functional properties, significantly influences tumor response to therapeutics. The heterogeneity of exosomes can lead to the acquisition of drug resistance in cancer cells through various mechanisms. For instance, TDEs can transfer drug resistance-conferring proteins, lipids, and nucleic acids, such as miRNAs, to sensitive cells, thereby spreading resistance within the TME^[4,11]^. Additionally, exosomes from CAFs have been shown to modulate the TME, promoting a phenotype that favors chemoresistance and conferring a survival advantage to cancer cells^[107]^. The heterogeneity of exosomes also presents challenges in their isolation and analysis, which is crucial for understanding their role in drug resistance. Standardization of isolation techniques, such as differential ultracentrifugation and size-exclusion chromatography, is essential for obtaining pure and consistent exosome preparations^[108]^. To address the impact of exosome heterogeneity on tumor drug resistance, a multifaceted approach is warranted. First, an in-depth study of the exosome biogenesis and cargo loading mechanism is necessary to help reveal the molecular mechanisms of how they mediate drug resistance. Second, the development of strategies to selectively target and neutralize exosomes carrying resistance-promoting cargo is crucial. This could involve the use of exosome inhibitors or the design of exosome mimetics that compete with TDEs for uptake by recipient cells. Third, leveraging the natural tumor-tropic properties of exosomes, researchers can engineer exosomes to deliver therapeutic agents directly to cancer cells, potentially reversing drug resistance^[87,109]^.

Moreover, the exact molecular determinants that dictate the selective packaging and delivery of resistance-inducing factors remain to be fully elucidated. The dynamics of exosome secretion and uptake in the context of evolving therapeutic pressures and their impact on treatment outcomes also require further investigation.

Here, we propose several avenues for future research to address these gaps. First, the development of high-throughput techniques for the comprehensive profiling of exosomal cargoes at different stages of disease and under varying therapeutic regimes will enhance our ability to identify signature biomarkers predictive of treatment response. Second, elucidating the molecular machinery governing exosome biogenesis, sorting, and release, particularly in drug-resistant cells, may uncover novel therapeutic targets. Third, leveraging the natural tropism of exosomes for specific cell types and their ability to cross biological barriers presents a unique opportunity to design exosome-based therapeutics to reverse resistance mechanisms or deliver targeted therapies. Finally, exploring the potential of exosome interception or engineering exosomes to express therapeutic payloads holds promise for restoring sensitivity to conventional treatments and enhancing patient outcomes. Collectively, these endeavors aim to transform our strategic approach toward combating drug resistance in cancer, paving the way for advancements in precision oncology.

## DECLARATIONS

### Copyright

© The Author(s) 2024.
